# Gastrointestinal foreign bodies in pet pigs: 17 cases

**DOI:** 10.1111/jvim.16429

**Published:** 2022-04-28

**Authors:** Yoko Nakamae, Kallie J. Hobbs, Jessie Ziegler, Luis A. Rivero, Shari Kennedy, Jenna Stockler, Diego E. Gomez

**Affiliations:** ^1^ Department of Clinical Studies, Ontario Veterinary College University of Guelph Guelph Ontario Canada; ^2^ Department of Large Animal Clinical Sciences University of Florida College of Veterinary Medicine Gainesville Florida USA; ^3^ Department of Veterinary Medical Teaching Hospital University of California School of Veterinary Medicine Davis California USA; ^4^ Department of Large Animal Clinical Sciences University of Missouri College of Veterinary Medicine Columbia Missouri USA; ^5^ Department of Clinical Sciences Auburn University College of Veterinary Medicine Auburn Alabama USA

**Keywords:** exploratory laparotomy, mortality, obstipation, survival, vomiting

## Abstract

**Background:**

Pigs have an indiscriminate eating behavior placing them at high risk of developing foreign body (FB) obstructions.

**Objectives:**

Describe the clinical and diagnostic features, treatments, and outcome of pet pigs diagnosed with gastrointestinal (GI) FBs. Medical and surgical treatments, pig outcomes, and post‐mortem findings were also investigated.

**Animals:**

Seventeen pet pigs.

**Methods:**

A multicenter retrospective study was conducted. Gastrointestinal FBs were defined as swallowed objects that became lodged within the gastrointestinal tract distal to the cardia identified during exploratory laparotomy.

**Results:**

Common clinical signs were anorexia/hyporexia, tachypnea, vomiting, dehydration, tachycardia, and ileus. Diagnostic imaging identified the presence of a FB in 4 cases. Upon celiotomy, the FBs were in the stomach and small intestine in 17 cases and large colon in 2 cases. Types of FB included fruit pit, diaper, and metallic objects. Of the 17 pigs, 15 (88%) were discharged from the hospital and 2 (12%) were euthanized.

**Conclusion and Clinical Importance:**

Clinical signs of GI FB were similar to those reported in obstipated pigs. Diagnostic imaging has limitations for detection of FB. Surgical removal of FBs in pigs carried a good prognosis.

**Abbreviations**
FB: foreign body
GI: gastrointestinal
GIT: gastrointestinal tract

## 1 INTRODUCTION

In recent decades, pet pigs have become popular in North America, resulting in a markedly increased demand of veterinary care at referral hospitals.^1^, ^2^, ^3^, ^4^ Pigs have a nonselective eating behavior, can swallow incompletely masticated food, and are exposed to human foodstuffs placing them at high‐risk of developing gastrointestinal (GI) foreign bodies (FBs). Gastrointestinal FB obstructions are commonly encountered in small animal medicine and are life‐threatening conditions; this disease appears to be uncommon or underreported in pigs.^5^ Swallowed FBs can be transported uneventfully through the gastrointestinal tract (GIT) although some FBs can cause intestinal obstruction requiring surgical removal of the offending object. Clinical signs of FBs such as vomiting, anorexia, and lethargic behavior can often mimic obstipation in pigs.^3^ Therefore, the objective of this retrospective multicenter study was to describe the clinical and diagnostic features of pet pigs diagnosed with GI FBs. Medical and surgical treatments, pig outcomes, and post‐mortem findings were also investigated.

## 2 MATERIALS AND METHODS

Medical records of pigs admitted to 4 veterinary teaching hospitals from North America, University of Florida (UF), University of Missouri (MU), Auburn University (AU) and University of California (UCD), between 2013 and 2019 were reviewed. Inclusion criteria included pigs of all ages and breeds and the presence of GI FBs (swallowed objects) that became lodged within the GIT distal to the cardia that was identified during exploratory laparotomy.

Information collected was: demographics, reproductive status, treatments and diagnostics performed before admission, presenting complaint, and time since last defecation. Physical examination findings, medical comorbidities, CBC and biochemistry profile (BP) results, and diagnostic imaging modality and findings were also recorded when available. Information regarding surgical intervention and intra‐operative findings, medical treatments, duration of hospitalization, and survival to discharge was recorded. For non‐surviving pigs, post‐mortem findings were registered. Descriptive statistics were reported using median and range values.

## 3 RESULTS

### 3.1 Study sample

Records from 779 pigs presented to the University of Florida (UF), 1627 pigs to the University California, 294 to the University of Missouri (MU), and 532 to Auburn University (AU) for evaluation and treatment of different conditions were reviewed. A total of 17 pigs (UC = 9, UF = 5, MU = 2 and AU = 1) met the inclusion criteria. Of the 17 cases, 9 were potbellied pigs, 3 were Juliana pigs, and 3 were mixed‐breed pigs. Median age was 3 years (range, 1‐8 years). There were 7 (41%) females and 10 (59%) males. Of these pigs, 6 were castrated males and 2 were spayed females. Presenting complaints included anorexia/hyporexia (n = 17, 100%), vomiting (n = 13, 76%), and absence of fecal output for >24 hours (n = 6, 35%). Reported median time since last defecation was 60 hours (range, 12‐168 hours), with 5 of 17 pigs having not defecated for ≥48 hours. Ingestion of a FB was observed by owners in 6 pigs (35%). Five pigs received medical treatment before admission including oral fluids (n = 1), enemas (n = 1), non‐steroidal anti‐inflammatories (NSAIDs; n = 2), antimicrobial drugs (n = 3), and antiemetics (n = 1).

### 3.2 Physical examination findings

On presentation, all 17 pigs were anorexic or hyporexic, 6 pigs vomited during physical examination, and 4 pigs had signs of colic (ie, teeth grinding, abdominal kicking, listless behavior). Median heart rate (HR) was 98 beats per minute (bpm; range, 80‐160 bpm). Nine (53%) pigs were tachycardic with a HR > 90 bpm. Median respiratory rate (RR) was 30 respirations per minute (rpm; range, 12‐96 rpm). Fifteen (88%) pigs were tachypneic with a RR > 20 rpm. Decreased borborygmi was noted in 9 (53%) pigs. Hydration status based on clinician assessment was available for 13 pigs. Of those, 11 pigs (65%) were reported to be dehydrated. Abdominal palpation was performed in 11 pigs, and 4 pigs exhibited pain. Three pigs had mild to moderate abdominal distention.

### 3.3 Clinicopathological findings

The CBC and BP results were available for 10 and 8 pigs, respectively. Frequent hematologic abnormalities consisted of leukopenia (<11 000 cells/μL) in 7 (41%) pigs and lymphopenia (<5300 cells/μL) in 9 (52%) pigs.^6^ The most common biochemical alteration was hyperglycemia (>120 mg/dL) in 3 (38%) of 8 pigs.^6^ Overall, hematologic and biochemical alterations were non‐specific.

### 3.4 Diagnostic imaging

Radiographic examination of the abdomen was performed in 14 (82%) pigs. The stomach was severely distended with homogenous, soft tissue opaque material and gas in 11 (65%) pigs. Distention was subjectively graded by deviation from normal placement and comparison to radiographic findings in pigs of similar weight. Gas‐distended small intestinal loops were noted in 9 (53%) pigs. A FB was identified in 4 (24%) cases. A linear metallic FB was noted in the stomach of 1 pig and 2 suspected peach pits were noted in the pylorus in another pig. Foreign material causing pyloric obstruction was described in the other 2 pigs. A dilated fluid‐filled stomach with gastric foreign material was confirmed in 1 pig, which elicited concerns of a pyloric outflow obstruction.

Ultrasonography findings were available for 6 (35%) pigs. In 3 cases, multiple hypomotile small intestine loops were noted. A mass‐like object with hypoechoic and hyperechoic regions was identified in the right ventral abdomen of a pig. A FB‐like material was identified in the pyloric region in another pig.

### 3.5 Treatment

Fourteen pigs (82%) were treated with fluid therapy via intravenous, rectal or oral, or combination of intravenous and oral administration of fluids, or combination of rectal and oral administration of fluids. Ten pigs received intravenous constant rate infusions at rates ranging from 2 to 6 mL/kg/h (median: 2.5 mL/kg/h) consisting of isotonic crystalloid fluids (n = 4), balanced electrolyte isotonic fluids (n = 5), or 5 isotonic crystalloid fluids mixed with 5% dextrose (n = 4). Duration of IV fluid therapy ranged from 24 to 72 hours (median: 48 hours). Rectal fluid administration rates ranged from 3 to 15 mL/kg/h (median: 11 mL/kg/h). The type of rectal fluids administered includes isotonic fluids (n = 2) or balanced electrolyte isotonic fluids (n = 3). Duration of rectal fluid therapy ranged from 24 to 48 hours (median: 24 hours). Six pigs were offered free choice access to water or fruit juice or both.

Sixteen (94%) pigs were administered antimicrobial drugs during hospitalization. Antimicrobial selection and dosing protocol were clinician‐dependent and varied across cases. Antimicrobial drugs included cephalosporins (n = 8), beta‐lactams (n = 8), tetracyclines (n = 1), macrolides (n = 1), and aminoglycosides (n = 1). Median duration of antimicrobial therapy was 4 days (range, 1‐14 days). Sixteen (94%) pigs were treated with NSAIDs including flunixin meglumine (14), meloxicam (2), and carprofen (4). Median duration of NSAID therapy was 4 days (range, 1‐6 days). Additional treatments administered included the antiemetic maropitant (n = 3) and gastroprotectants (ie, sucralfate, proton pump inhibitors; n = 8). Fourteen (83%) pigs were initially treated medically but failed to respond and an exploratory laparotomy was performed.

### 3.6 Surgical intervention

All pigs underwent exploratory laparotomy. Surgical procedures performed included gastrotomy (5), enterotomy (5), typhlotomy (1), or a combination of gastrotomy and enterotomy (5). In 1 pig with marked peritonitis secondary to duodenal rupture from a peach pit an exploratory laparotomy was the only surgical procedure performed. Nineteen FBs were detected in the 17 pigs and were in the stomach (n = 9, 47%), small intestine (n = 8, 42%), colon (n = 1, 5.5%), and cecum (n = 1, 5.5%). Pigs with >1 FB had 1 FB in the stomach and the other 1 located in the small intestine. In 1 pig, 3 FBs (foam bedding) were removed from the stomach (pylorus) and proximal duodenum. Types of FBs included fruit pit (n = 6, 35%), diaper (n = 3, 18%), foam (2, 12%), metallic objects (n = 2, 12%), bezoars (n = 2, 6%), and glass fragments (n = 2, 6%). In 1 case, rope‐like FB was found in multiple locations. Of these fruit pits, peach pit was commonly observed. In 1 case, a rope‐like FB was found in multiple locations. Gastric impaction (n = 2), cecal impaction (n = 1), spiral colon impaction (n = 1), small intestine impaction (n = 1), and jejunal rupture with peritonitis (n = 1) were also noted during surgery. In 1 pig, a perforating duodenal FB (peach pit) with marked peritoneal contamination of fecal and feed material was noted. Information regarding the time of admission to surgery was not collected. Of the 17 pigs, 15 (88%) were discharged from the hospital and 2 (12%) were euthanized during surgery because of severe peritonitis (n = 1) and poor viability of the jejunum with the presence of a small rupture and peritonitis (n = 1).

### 3.7 Complications

Three pigs developed complications. One pig had mild intraoperative contamination of the abdominal cavity that was treated with intravenous antimicrobial drugs and responded well to therapy (FB in cecum). In another pig, a soft tissue abscess developed in the caudal aspect of the incision at 30 days post‐surgery (FB in stomach). The abscess was debrided surgically under sedation. One pig developed constipation 2 weeks later (FB at pyloric outflow), which resolved without further treatment.

### 3.8 Outcome

Fifteen (88%) pigs were discharged from the hospital while 2 (12%) were euthanized during surgery because of the poor prognosis. The median time of hospitalization for pigs that survived to discharge was 4 days (range, 2 to 8 days). The median time between admission and euthanasia in non‐surviving pigs was 18 hours (range, 0.5‐24 hours). Long‐term follow‐up was not available for pigs discharged from the hospital.

### 3.9 Post‐mortem examination findings

Post‐mortem examination was performed in the 2 euthanized pigs. In 1 pig, locally extensive and acute duodenal perforation coupled with mild–to‐moderate peritoneal effusion and peritonitis were noted. In the other pig, the stomach contained a light green to yellow liquid and a 3.4 × 2.4 × 2–cm^3^ peach pit was present in the fundus of the stomach. The jejunal and duodenal regions were fluid filled with a dark‐red to black color transmurally with a jejunal perforation.

## 4 DISCUSSION

In this study, the most frequently observed clinical findings were anorexia or hyporexia, tachypnea, vomiting, dehydration, hypomotility, tachycardia, ileus, and decreased demeanor. These clinical signs are similar to those reported in pigs with obstipation.^3^ Therefore, clinicians should always consider FB obstruction as a differential diagnosis for pigs with decreased fecal output, anorexia or vomiting.

The FBs in our study were primarily identified within the stomach and small intestine and in most of the cases were causing a complete obstruction requiring surgical intervention. Foreign bodies located in the jejunum, spiral colon, transverse, and descending colon of pigs have been reported, but are noted as a rare occurance.^5^ Studies in dogs investigating FBs located distal to the cardia showed contradictory results with some reporting that most of the obstructions occurred in the stomach^7^ and others with predominant occurrence in the jejunum.^8^, ^9^ Of the FBs identified distal to the cardia in dogs, 70% caused complete obstruction.^8^ It is possible that FB obstruction of the stomach and small intestine in pigs are more frequent because FBs that pass through the small diameter of the small intestine are then able to freely move distally without causing clinical signs of obstruction.

Causes of intestinal obstructions included fruit pits, diapers, and linear foreign bodies. Fruit pits and linear FBs have previously been reported to cause GI obstruction in pigs.^5^ In small animals, linear FBs account for 50% to 60% of the cases in cats, and 36% in dogs. The survival rate of dogs with linear FBs varies from 78% to 98%.^7^, ^8^ Dogs with linear FBs had more severe clinical signs and an increased duration of hospitalization than their counterparts with non‐linear FBs.^8^, ^10^ In our study, hospitalization period and survival rates appeared to be similar for pigs with linear and non‐linear FBs. However, the small number of pigs included in this study did not allow statistical analysis to determine differences between groups.

Esophageal and gastric endoscopic FB removal is reported in humans,^11^ dogs,^8^, ^9^ calves,^12^ horses,^13^ and donkeys.^14^ Flexible endoscopy allows direct observation of the foreign material, facilitates its removal, and allows assessment of stomach integrity. Success rates for endoscopic removal of esophageal and gastric FBs in dogs ranged from 26% to 63% in early studies, however, a more recent study reported a success rate of 86% (57/66).^9^ The value of endoscopic FB removal in pigs is unknown. In our study, there were no attempts to remove gastric FBs. In our study, sample 65% of the pigs had a severely distended stomach with homogenous soft tissue opaque material on radiographic examination of the abdomen, which may limit accessibility to objects located near or distal to the pylorus. Endoscopic FB removal could be beneficial in cases with a discrete FB as it is less invasive and can reduce the risk of general anesthesia or other complications associated with laparotomy (eg, peritonitis, incision infections, seromas, and abscesses).^5^, ^8^

Gastric FBs were successfully removed via gastrostomy or enterotomy with only 3 (20%) of 15 pigs developing complications (intraoperative abdominal contamination, surgical site abscess, and constipation). Generally, in small animals, gastrotomy is a routine procedure with an excellent prognosis and minimal complications, and dehiscence and peritonitis rarely occur if basic surgical rules are followed.^8^, ^15^ Our study indicates that this statement could also apply in pet pigs. In general, the enterotomy procedure itself is not associated with higher fatality rate or complications in dogs.^8^ However, dogs and pigs undergoing multiple intestinal incisions have a significantly higher fatality rate than those treated with a single enterotomy.^5^, ^8^ The number of incisions was not recorded in the present study and therefore further conclusions cannot be drawn.

Gastrointestinal FBs were identified in 24% of pigs via radiographic examination. In small animals, ultrasonography detects 100% of FBs in animals, while radiographs only identify 56% of foreign bodies.^16^ Ultrasonography aids in identifying intestinal perforation, serosal alterations, and mild small intestinal distension, but these abnormalities cannot be identified using radiographic examination.^16^ Abdominal ultrasonography findings were reported in 6 pigs in our study, with the FB identified in 1 pig. Differences between human and pig medicine regarding the diagnostic value of abdominal ultrasonography can be explained, at least in part, by the difficulties in clearly identify abdominal structures because of their thick skin and greater abdominal fat layer. Computed tomography (CT) for evaluation of the GIT is commonly used in small animals and humans for diagnosis of GI diseases.^17^ There is growing acceptance for its use in pigs for the diagnosis of GI diseases but is often declined for economic reasons. The CT examination could aid in diagnosis of GI FBs when the diagnosis is not reached by radiography or ultrasonography examination.

Surgical removal of FBs in pigs included in this study carried a good prognosis for survival. A previous report documented a successful removal of FBs in 5 of 5 pigs with intestinal obstruction.^5^ The survival proportion of the pigs reported here is similar to the 88% and 92% survival proportions reported in cats and dogs,^8^ respectively, undergoing celiotomy for removal of FBs located distal to the cardia.^8^

Limitations of this study include those associated with the retrospective design. The small sample size of the different treatment groups also prevented the assessment of risk factors for survival and the efficacy of the different interventions. This study provides evidence that surgical removal of FBs has a good prognosis, and that FBs need to be considered as a differential diagnosis in pigs with clinical signs of vomiting, anorexia, lack of fecal passage, or lethargic behavior.

## CONFLICT OF INTEREST DECLARATION

Authors declare no conflict of interest.

## OFF‐LABEL ANTIMICROBIAL DECLARATION

Authors declare no off‐label use of antimicrobials.

## INSTITUTIONAL ANIMAL CARE AND USE COMMITTEE (IACUC) OR OTHER APPROVAL DECLARATION

Authors declare no IACUC or other approval was needed.

## HUMAN ETHICS APPROVAL DECLARATION

Authors declare human ethics approval was not needed for this study.
