# Erratum for “Gastrointestinal foreign bodies in pet pigs: 17 cases”

**DOI:** 10.1111/jvim.16475

**Published:** 2022-06-23

First published: April 28, 2022 https://onlinelibrary.wiley.com/doi/10.1111/jvim.16429

Volume 36, Issue 3.

Correction to Off‐label Antimicrobial Declaration as follows.

Authors declare off‐label use of antimicrobials. Cephalosporins, oxytetracycline, penicillin, macrolides, and aminoglycosides used in the pigs included in this article are not labelled for surgical prophylaxis or similar indications in the United States

